# Supplementary material for: Polygenic risk score for ulcerative colitis predicts immune checkpoint inhibitor-mediated colitis
Source: Nat Commun. 2024 Mar 26;15:2568. doi: 10.1038/s41467-023-44512-4 (PMC10966072; doi:10.1038/s41467-023-44512-4)
Supplement: Supplementary file 1 — Supplementary Information [file 41467_2023_44512_MOESM1_ESM.pdf]

Supplementary Information

Polygenic risk score for ulcerative colitis predicts immune checkpoint inhibitor-mediated colitis

Pooja Middha[1], Rohit Thummalapalli[2], Michael J. Betti[3], Lydia Yao[4], Zoe Quandt[5,6], Karmugi Balaratnam[7], Cosmin A. Bejan[8], Eduardo Cardenas[1], Christina J. Falcon[9], David M. Faleck[10], Princess Margaret Lung Group[7], Matthew A. Gubens[11,12], Scott Huntsman[1], Douglas B. Johnson[13], Linda Kachuri[14,15], Khaleeq Khan[7], Min Li[1], Christine M. Lovly[16], Megan H. Murray[4], Devalben Patel[7], Kristin Werking[17], Yaomin Xu[4], Luna Jia Zhan[7], Justin M. Balko[13], Geoffrey Liu[7,18,19], Melinda C. Aldrich[3], Adam J. Schoenfeld[20], Elad Ziv[1,21,22,23]

[1] Department of Medicine, University of California San Francisco, San Francisco, CA, USA

[2] Department of Medicine, Memorial Sloan Kettering Cancer Center, New York, NY, USA

[3] Department of Medicine, Division of Genetic Medicine, Vanderbilt University Medical Center, Nashville, TN, USA

[4] Department of Biostatistics, Vanderbilt University Medical Center, Nashville, TN, USA

[5] Division of Endocrinology and Metabolism, Department of Medicine, University of California San Francisco, San Francisco, CA, USA

[6] Diabetes Center, University of California San Francisco, San Francisco, CA, USA

[7] Princess Margaret Cancer Centre, Toronto, ON, Canada

[8] Department of Biomedical Informatics, Vanderbilt University Medical Center, Nashville, TN, USA

[9] Fiona and Stanley Druckenmiller Center for Lung Cancer Research, Memorial Sloan Kettering Cancer Center, New York, NY, USA

[10] Gastroenterology, Hepatology & Nutrition Service, Department of Medicine, Memorial Sloan Kettering Cancer Center, New York, NY, USA

[11] Medical Oncology, University of California San Francisco, San Francisco, CA, USA

[12] Department of Medicine, Weill Cornell Medical Center, New York, NY, USA

[13] Department of Medicine, Vanderbilt University Medical Center, Nashville, TN, USA.

[14] Department of Epidemiology and Population Health, Stanford University School of Medicine, Stanford, CA, USA

[15] Stanford Cancer Institute, Stanford University of Medicine, Stanford, CA, USA

[16] Department of Medicine, Division of Hematology and Oncology, Vanderbilt University Medical Center and Vanderbilt Ingram Cancer Center, Nashville, TN, USA

[17] Department of Thoracic Surgery, Vanderbilt University Medical Center, Nashville, TN, USA

[18] Temerty School of Medicine, , Toronto, ON, Canada

[19] Dalla Lana School of Public Health, University of Toronto, Toronto, ON, Canada

[20] Thoracic Oncology Service, Memorial Sloan Kettering Cancer Center, New York, NY, USA

[21] Helen Diller Family Comprehensive Cancer Center, University of California San Francisco, San Francisco, CA, USA

[22] Center for Genes, Environment and Health, University of California San Francisco, San Francisco, CA, USA

[23] Institute for Human Genetics, University of California San Francisco, San Francisco, CA, USA

Corresponding Author
Dr. Elad Ziv (Elad.Ziv@ucsf.edu)

Supplementary Table 1: Characteristics of the entire GeRI cohort and by recruiting site

Characteristics	GeRI	MSKCC	PM	UCSF	VUMC
	(n=1316)	(n=752)	(n=266)	(n=31)	(n=267)
Mean age at diagnosis (SD)	65.24 (10.26)	66.13 (10.52)	64.60 (10.48)	65.30 (9.71)	63.33 (9.01)
Sex n (%)					
Male	659 (50.1)	353 (46.9)	142 (53.4)	12 (38.7)	152 (56.9)
Female	657 (49.9)	399 (53.1)	124 (46.6)	19 (61.3)	115 (43.1)
Self-reported race					
White	914 (69.5)	469 (64.0)	167 (62.8)	31 (100)	246 (92.1)
Asian	89 (6.8)	34 (4.6)	46 (17.3)	-	2 (0.7)
Black	70 (5.3)	41 (5.6)	10 (3.8)	-	14 (5.2)
Other	16 (1.2)	20 (0.2)	52 (4.4)	-	3 (1.1)
Missing	227 (17.2)	188 (25.6)	31 (11.7)	-	2 (0.7)
Histology n (%)					
Adenocarcinoma	949 (72.1)	580 (77.1)	193 (72.6)	24 (77.4)	152 (56.9)

Squamous cell carcinoma	221 (16.8)	111 (14.8)	46 (17.3)	5 (16.1)	59 (22.1)
Other	146 (11.1)	61 (8.1)	27 (10.2)	2 (6.5)	56 (21.0)
Type of therapy n (%)					
Anti PD-1/PD-L1 therapy	1198 (91.0)	671 (89.2)	257 (96.6)	31 (100.0)	239 (89.5)
Anti PD-1/PD-L1 + Anti CTLA4 therapy	118 (9.0)	81 (10.8)	9 (3.4)	0 (0.0)	28 (10.5)
IMC n (%)					
Yes	55 (4.2)	32 (4.3)	13 (4.9)	1 (3.2)	9 (3.4)
No	1261 (95.8)	720 (95.7)	253 (95.1)	30 (96.8)	258 (96.6)
Mean time in months to IMC (SD)	9.85 (13.37)	9.78 (13.42)	11.07 (13.75)	15.28 (-)	8.19 (11.77)
Severe IMC* n (%)					
Yes	32 (2.4)	15 (2.0)	10 (3.8)	1 (3.2)	6 (2.2)
No	1261 (95.8)	720 (95.7)	253 (95.1)	30 (96.8)	258 (96.6)
Progression n (%)					
Yes	999 (75.9)	640 (85.1)	177 (66.5)	22 (71.0)	160 (59.9)
No	315 (23.9)	112 (14.9)	89 (33.5)	8 (25.8)	106 (39.7)

Missing	2 (0.2)	0 (0.0)	0 (0.0)	1 (3.2)	1 (0.4)
Mean time in months to progression (SD)	10.02 (13.44)	10.11 (13.68)	9.67 (11.13)	27.07 (23.67)	8.18 (11.74)
Overall survival n (%)					
Alive at last follow-up	410 (31.2)	262 (34.8)	131 (49.2)	17 (54.8)	0 (0.0)
Deceased	639 (48.6)	490 (65.2)	135 (50.8)	14 (45.2)	0 (0.0)
Missing	267 (20.3)	0 (0.0)	0 (0.0)	0 (0.0)	267 (100.0)
Mean time in months to death (SD)	22.09 (17.81)	21.71 (16.99)	20.84 (18.57)	43.37 (18.88)	-

* The combined percentage does not add to 100 because mild-to-moderate grade IMC were excluded from this grouping. MSKCC, Memorial Sloan Kettering Cancer Center; PM, Princess Margaret Cancer Centre; UCSF, University of California San Francisco; VUMC, Vanderbilt University Medical Center; SD: Standard deviation; IMC: Immune checkpoint inhibitor-mediated colitis

Supplementary Table 2: Characteristics of the BioVU replication cohort overall and by immune checkpoint inhibitor-mediated colitis (IMC)

Characteristics	Overall	All-grade IMC	Severe IMC
	(n=1147)	(n=104)	(n=83)
Mean age at diagnosis (SD)	62.1 (13.1)	61.6 (12.7)	61.4 (12.9)
Sex, n (%)			
Male	727 (63.4)	62 (59.6)	48 (57.8)
Female	420 (36.6)	42 (40.4)	35 (42.2)
Type of therapy, n (%)			
Anti PD-1/PD-L1 monotherapy	828 (72.7)	34 (32.7)	23 (27.7)
Anti PD-1/PD-L1 + Anti CTLA4 therapy	45 (3.9)	25 (24.0)	22 (26.5)
Anti CTLA4 monotherapy	274 (23.9)	45 (43.3)	38 (45.8)

IMC: Immune checkpoint inhibitor-mediated colitis, SD: Standard deviation

Supplementary Table 3: Previously published polygenic risk score (PRS) of ulcerative colitis as a predictor of time to development of all-grade immune checkpoint inhibitor-mediated colitis (IMC) in the entire GeRI cohort, using Cox proportional hazards models

All-grade IMC					
PRS[a]	PRS method	HR per SD	95% CI	P	Ref.
179-SNP PRS	SNPnet	1.24	0.94 - 1.64	0.13	[1]
809-SNP PRS	SNPnet	1.33	1.01 – 1.74	0.04	[1]
1,505-SNP PRS	Penalized Regression	1.37	1.02 – 1.85	0.04	[2]
566,637-SNP PRS	LDPred2	1.41	1.04 – 1.91	0.03	[2]

[a]Models are adjusted for age at diagnosis, sex, histology, type of therapy, recruiting site, and 5 principal components. IMC: Immune checkpoint inhibitor-mediated colitis, PRS: Polygenic risk score, HR: Hazard ratio, SD: Standard deviation, CI: Confidence interval, SNP: Single nucleotide polymorphism

Supplementary Figure 1: Predictive performance of (a) polygenic risk score of Crohn's disease (PRS_{CD}) and (b) polygenic risk score of ulcerative colitis (PRS_{UC}), respectively, using Area under the receiver operating curves (AUC) in the testing data (30% of UK Biobank: 394 Crohn's disease cases, 844 ulcerative colitis cases, and 4,891 controls).

(a) PRS_{CD} (b) PRS_{UC}

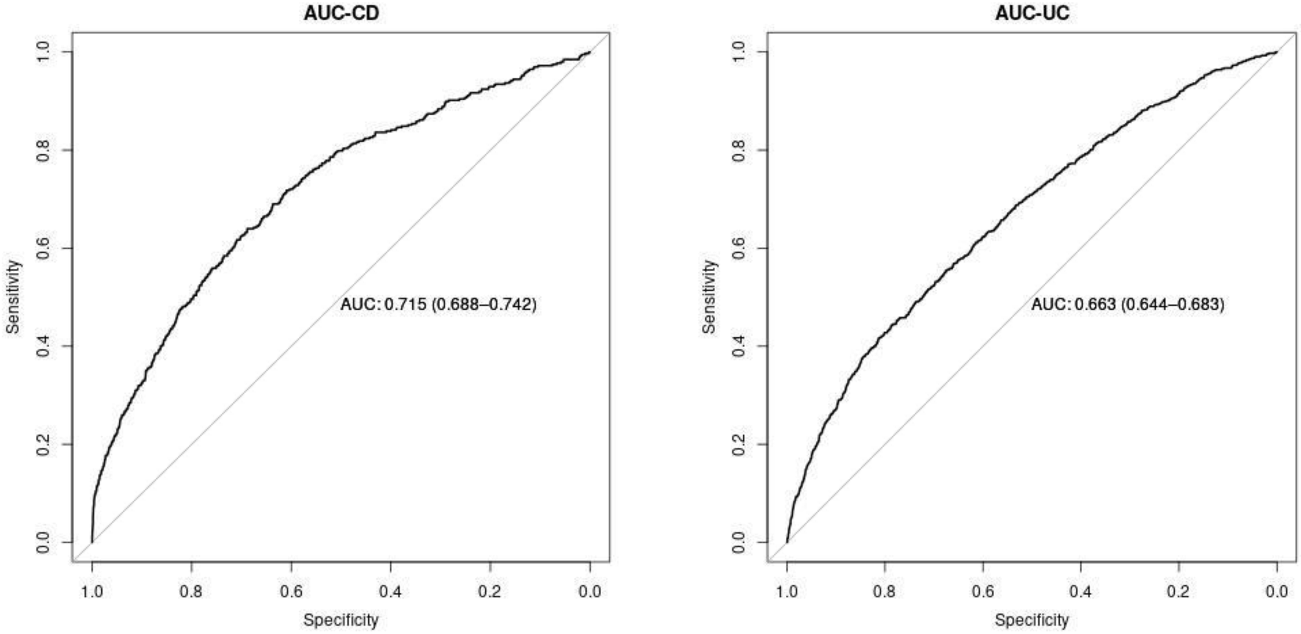

Supplementary Figure 2: Predictive performance of (a) polygenic risk score of Crohn's disease (PRS_{CD}) and (b) polygenic risk score of ulcerative colitis (PRS_{UC}), respectively, using Area under the receiver operating curves (AUC) in the validation set (cancer-free BioVU individuals: 1,420 Crohn's disease cases, 459 ulcerative colitis cases, and 20,876 controls).

(a) PRS_{CD}

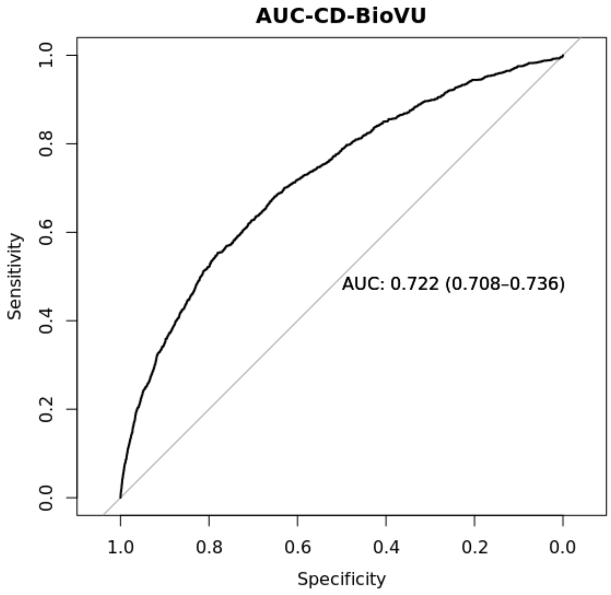

(b) PRS_{UC}

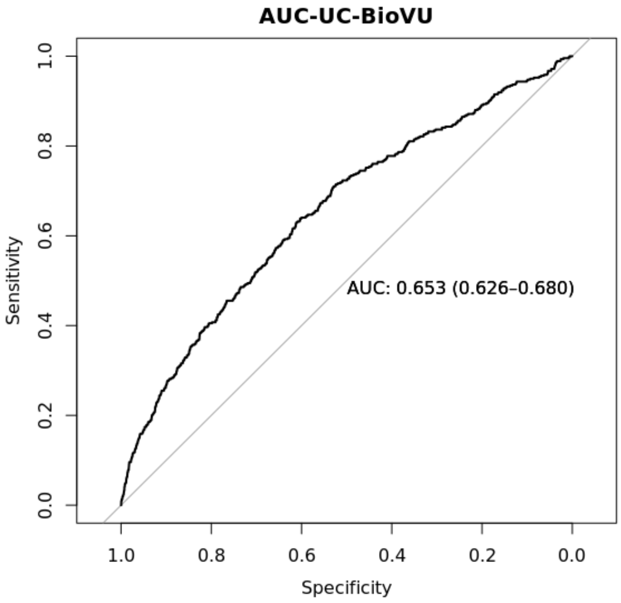

Supplementary Figure 3: Violin plots showing the distribution of the polygenic risk score of ulcerative colitis (PRS_{UC}) for (a) All-grade immune checkpoint inhibitor-mediated colitis (IMC) (55 cases) and (b) Severe IMC (32 cases) in the GeRI cohort. The box plots extend from the 25th to 75th percentiles. Statistical significance was determined by the T-test, and all p-values are two-sided. Source data are provided as a Source Data file.

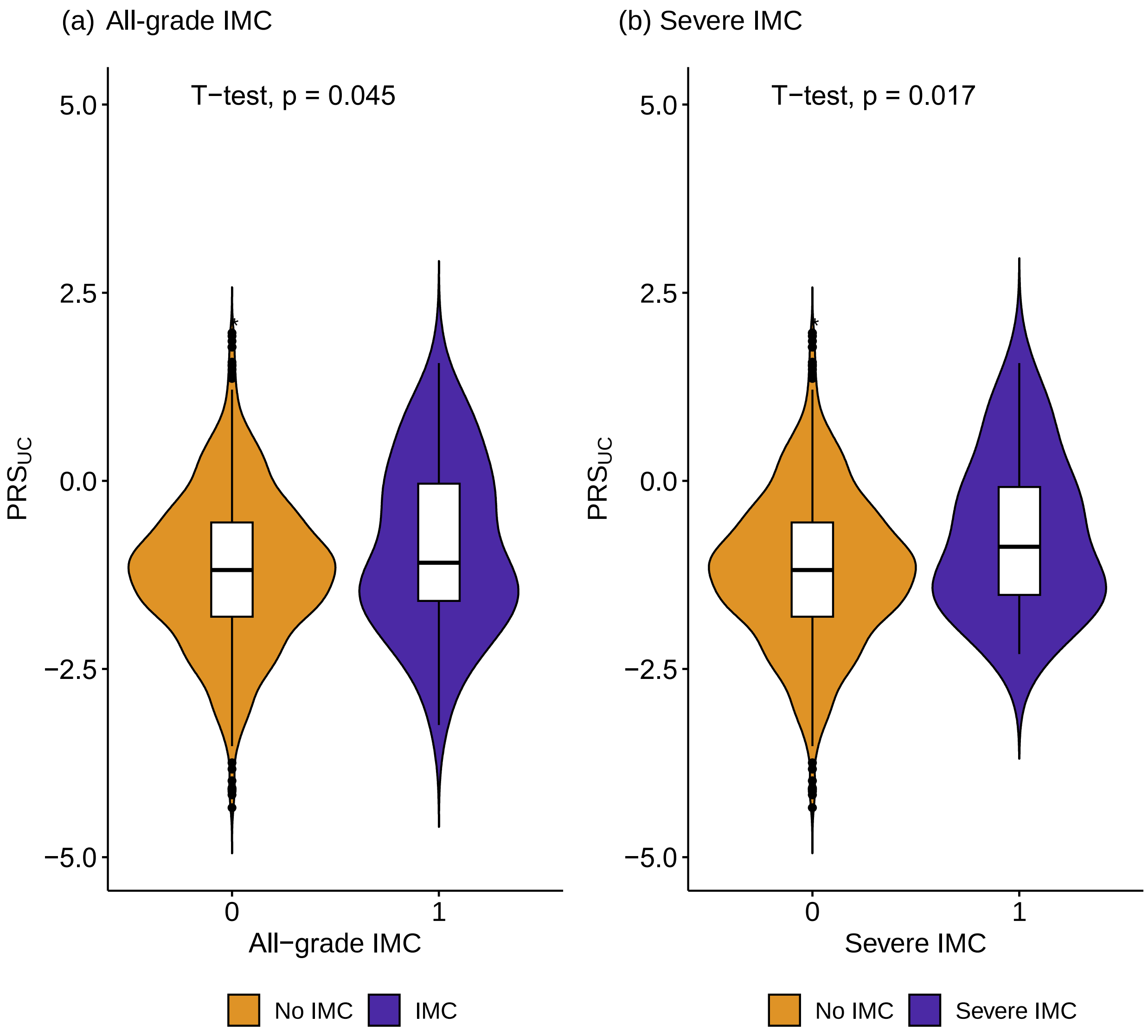

Supplementary Figure 4: Associations of known ulcerative colitis-associated HLA markers (frequency ≥0.01) with all-grade immune checkpoint inhibitor-mediated colitis (IMC) (55 cases) in the GeRI cohort. Logistic regression models were adjusted for age at diagnosis, sex, lung cancer histology, type of therapy, recruiting site, and 5 PCs. Source data are provided as a Source Data file.

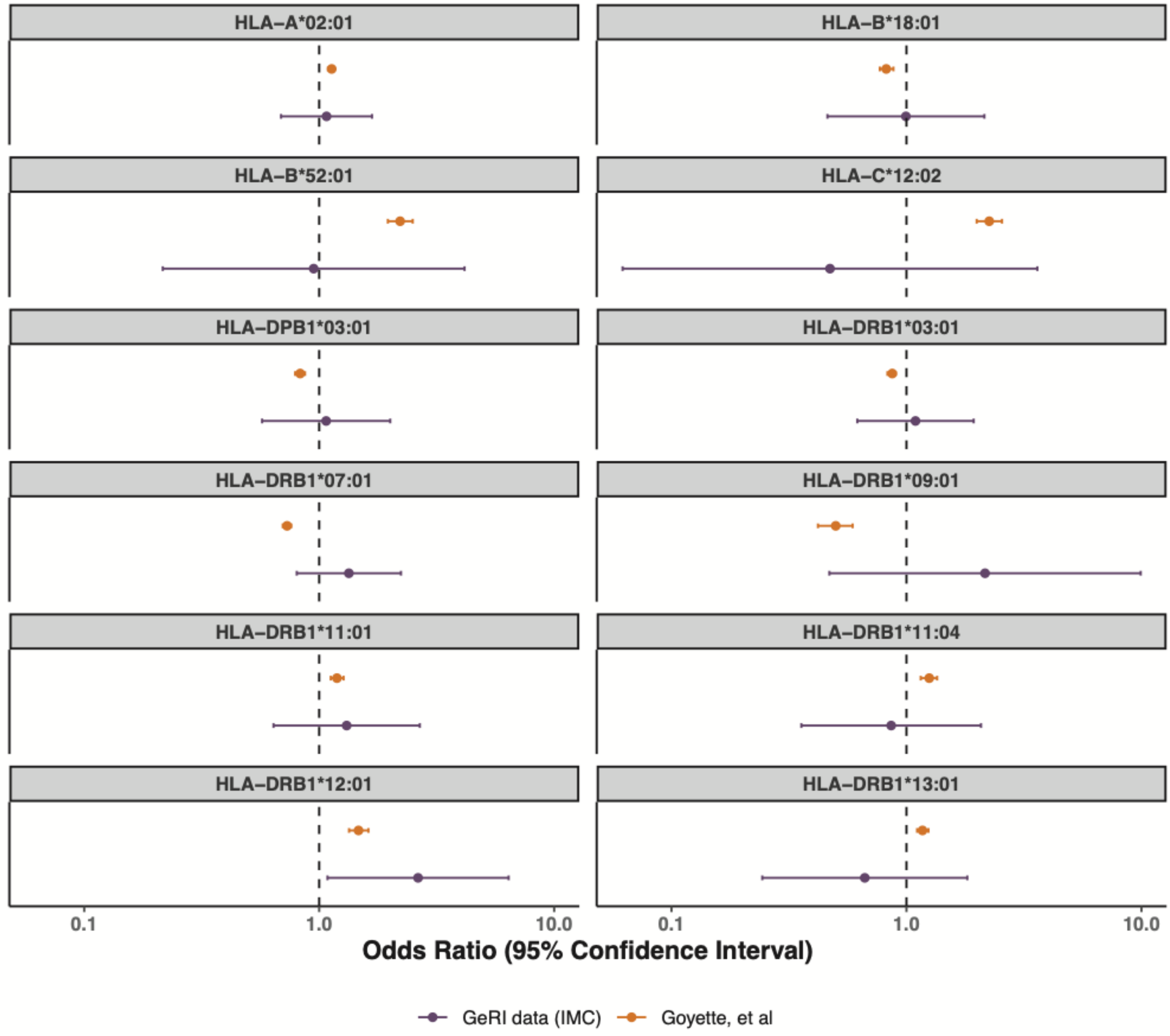

Supplementary Figure 5: Immune checkpoint inhibitor-mediated colitis (IMC) as a predictor of progression-free survival (PFS) in the GeRI cohort for (a) All-grade IMC and (b) Severe IMC. Kaplan–Meier survival curves are unadjusted with 90-day landmark and compare those who had an IMC (all-grade or severe) with those who did not have an IMC (No IMC). The p-values in the graph represent the log-rank p-values (two-sided) and the dotted line represents median survival time. Source data are provided as a Source Data file.

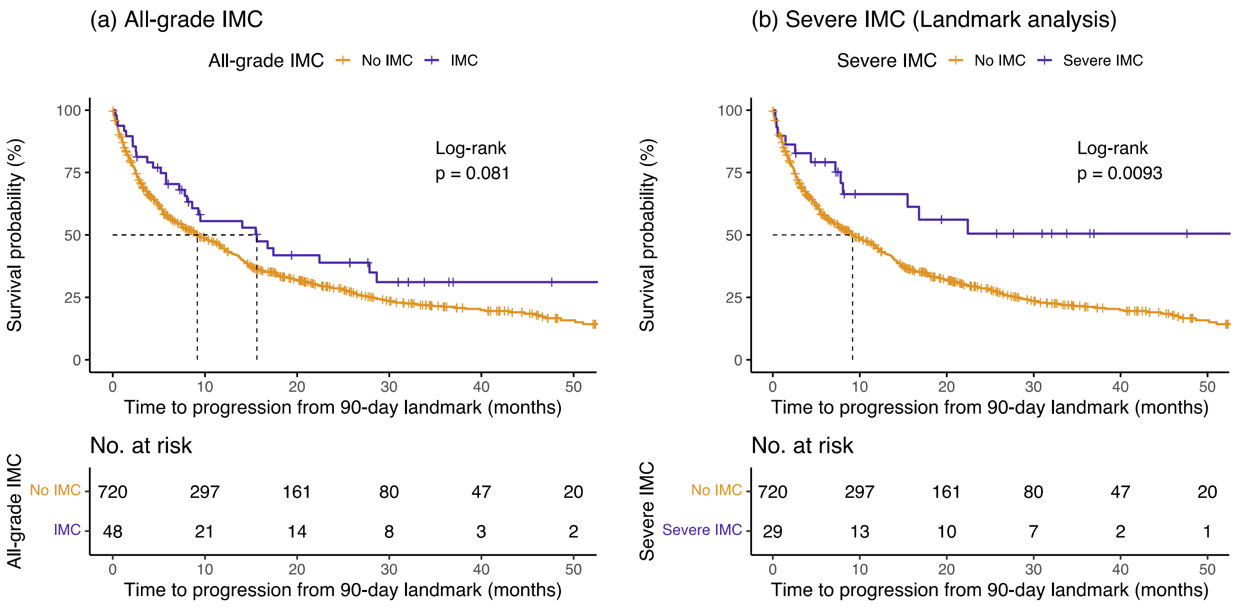

Supplementary References

1. Tanigawa Y, Qian J, Venkataraman G, et al. Significant sparse polygenic risk scores across 813 traits in UK Biobank. PLOS Genetics. 2022;18(3):e1010105. doi:10.1371/journal.pgen.1010105

2. Privé F, Aschard H, Carmi S, et al. Portability of 245 polygenic scores when derived from the UK Biobank and applied to 9 ancestry groups from the same cohort. The American Journal of Human Genetics. 2022;109(1):12-23. doi:10.1016/j.ajhg.2021.11.008
