# Supplementary material for: Polygenic risk score for ulcerative colitis predicts immune checkpoint inhibitor-mediated colitis
Source: Nat Commun. 2024 Mar 26;15:2568. doi: 10.1038/s41467-023-44512-4 (PMC10966072; doi:10.1038/s41467-023-44512-4)
Supplement: Supplementary file 3 — Reporting Summary [file 41467_2023_44512_MOESM3_ESM.pdf]

nature portfolio

Corresponding author(s): Elad Ziv

Last updated by author(s): Dec 13, 2023

Reporting Summary

Nature Portfolio wishes to improve the reproducibility of the work that we publish. This form provides structure for consistency and transparency in reporting. For further information on Nature Portfolio policies, see our Editorial Policies and the Editorial Policy Checklist.

Statistics

For all statistical analyses, confirm that the following items are present in the figure legend, table legend, main text, or Methods section.

n/a	Confirmed	
☐	☒	The exact sample size (*n*) for each experimental group/condition, given as a discrete number and unit of measurement
☒	☐	A statement on whether measurements were taken from distinct samples or whether the same sample was measured repeatedly
☐	☒	The statistical test(s) used AND whether they are one- or two-sided<br>*Only common tests should be described solely by name; describe more complex techniques in the Methods section.*
☐	☒	A description of all covariates tested
☐	☒	A description of any assumptions or corrections, such as tests of normality and adjustment for multiple comparisons
☐	☒	A full description of the statistical parameters including central tendency (e.g. means) or other basic estimates (e.g. regression coefficient) AND variation (e.g. standard deviation) or associated estimates of uncertainty (e.g. confidence intervals)
☐	☒	For null hypothesis testing, the test statistic (e.g. *F*, *t*, *r*) with confidence intervals, effect sizes, degrees of freedom and *P* value noted<br>*Give P values as exact values whenever suitable.*
☒	☐	For Bayesian analysis, information on the choice of priors and Markov chain Monte Carlo settings
☒	☐	For hierarchical and complex designs, identification of the appropriate level for tests and full reporting of outcomes
☐	☒	Estimates of effect sizes (e.g. Cohen's *d*, Pearson's *r*), indicating how they were calculated

Our web collection on statistics for biologists contains articles on many of the points above.

Software and code

Policy information about availability of computer code

Data collection	No software was used for data collection
Data analysis	We used R programming language (v4.2.2), survival R package (v3.4.0), ggplot2 package (v3.4.2), pROC package (v1.18.0), bigsnpr package (v1.11.6), and PLINK2 for PRS calculations. Analysis scripts can be found at https://github.com/PoojaMiddha/GeRI_colitis

For manuscripts utilizing custom algorithms or software that are central to the research but not yet described in published literature, software must be made available to editors and reviewers. We strongly encourage code deposition in a community repository (e.g. GitHub). See the Nature Portfolio guidelines for submitting code & software for further information.

Data

Policy information about availability of data

All manuscripts must include a data availability statement. This statement should provide the following information, where applicable:

- Accession codes, unique identifiers, or web links for publicly available datasets
- A description of any restrictions on data availability
- For clinical datasets or third party data, please ensure that the statement adheres to our policy

UK Biobank data are publicly available by request from https://www.ukbiobank.ac.uk. Scoring files for Crohn's disease and ulcerative colitis are available from the PGS catalog http://www.pgscatalog.org/score/PGS004253/ and http://www.pgscatalog.org/score/PGS004254/ . Deidentified data along with outcomes used for this work are available at https://github.com/PoojaMiddha/GeRI_colitis/tree/GeRI_colitis_manuscript. BioVU data is available to Vanderbilt-affiliated members subject to approval by the BioVU Review Committee from https://victr.vumc.org/how-to-use-biovu/. External Users will need a Vanderbilt PI for all BioVU projects, and

contracts will need to be in place before data can be shared. Investigators can reach out to BioVU (biovu@vumc.org) if they would like more information or help establishing a collaboration. The remaining data are available within the Article, Supplementary Information or Source Data file.

Research involving human participants, their data, or biological material

Policy information about studies with human participants or human data. See also policy information about sex, gender (identity/presentation), and sexual orientation and race, ethnicity and racism.

Reporting on sex and gender	- We had information on self-identified gender assigned at birth and included gender as a covariate.<br>- 49% of the participants were female and 51% male in the GeRI cohort.
Reporting on race, ethnicity, or other socially relevant groupings	Self-reported race and ethnicity was provided by small proportion of participants. However, we used principal components to account for population stratification as covariates in the analysis. Race and ethnicity were not included in the analyses.
Population characteristics	- The UK Biobank is a population-based prospective cohortf nearly 500,000 individuals aged 40-69 years from the United Kingdom.<br>- The Vanderbilt University Medical Center BioVU is a synthetic derivative biobank linked to de-identified electronic health records.<br>- GeRI cohort is a cohort of 1,316 advanced non-small cell lung cancer cases who have had at least one dose of immunotherapy as part of their standard of care. They are recruited from four centers: University of California San Francisco, Memorial Sloan Kettering Cancer Center, Vanderbilt University Medical Center, and Princess Margaret Cancer Center (Canada).
Recruitment	GeRI cohort is comprised of: Advanced Stage IIIB/IV NSCLC patients who received ICI therapy (PD-1 or PD-L1 inhibitors as monotherapy or in combination with either CTLA-4 inhibitors and/or chemotherapy).
Ethics oversight	Institutional Review Board approvals were obtained at each site individually and written informed consent was acquired from all study participants prior to inclusion in the study. UK Biobank got ethics approval from the Research Ethics Committee (REC reference: 11/NW/0382) in accordance with the UK Biobank Ethics and Governance framework.

Note that full information on the approval of the study protocol must also be provided in the manuscript.

Field-specific reporting

Please select the one below that is the best fit for your research. If you are not sure, read the appropriate sections before making your selection.

☒ Life sciences ☐ Behavioural & social sciences ☐ Ecological, evolutionary & environmental sciences

For a reference copy of the document with all sections, see nature.com/documents/nr-reporting-summary-flat.pdf

Life sciences study design

All studies must disclose on these points even when the disclosure is negative.

Sample size	1,316 advanced non-small cell lung cancer patients treated with at least one dose of immunotherapy with available genotype and phenotype data were used.
Data exclusions	No data was excluded from the analysis
Replication	The goal of the present analysis is to assess the clinical utility of polygenic risk score for ulcerative colitis and Crohn's disease to identify patients at high risk of developing immune-mediated colitis upon treatment with immune checkpoint inhibitor. The predictive power and validity of polygenic risk score of ulcerative colitis and Crohn's disease was tested in 30%of UK Biobank and BioVU.
Randomization	This is an observational genetic study and not randomized. Treatment with immunotherapy was part of the standard of care for the patients at each participating site.
Blinding	This is an observational genetic study, and not a clinical trial.

Reporting for specific materials, systems and methods

We require information from authors about some types of materials, experimental systems and methods used in many studies. Here, indicate whether each material, system or method listed is relevant to your study. If you are not sure if a list item applies to your research, read the appropriate section before selecting a response.

Materials & experimental systems

n/a	Involved in the study
☒	☐ Antibodies
☒	☐ Eukaryotic cell lines
☒	☐ Palaeontology and archaeology
☒	☐ Animals and other organisms
☒	☐ Clinical data
☒	☐ Dual use research of concern
☒	☐ Plants

Methods

n/a	Involved in the study
☒	☐ ChIP-seq
☒	☐ Flow cytometry
☒	☐ MRI-based neuroimaging
